# Varia: a tool for prediction, analysis and visualisation of variable genes

**DOI:** 10.1186/s12859-022-04573-6

**Published:** 2022-01-24

**Authors:** Gavin Mackenzie, Rasmus W. Jensen, Thomas Lavstsen, Thomas D. Otto

**Affiliations:** 1 Centre for Immunobiology, Institute of Infection, Immunity and Inflammation, MVLS, University of Glasgow, Glasgow, UK; 2 Department of Immunology and Microbiology, Department of Infectious Diseases, Centre for Medical Parasitology, University of Copenhagen, 1017 Rigshospitalet, Copenhagen, Denmark

## Abstract

**Background:**

Parasites use polymorphic gene families to evade the immune system or interact with the host. Assessing the diversity and expression of such gene families in pathogens can inform on the repertoire or host interaction phenotypes of clinical relevance. However, obtaining the sequences and quantifying their expression is a challenge. In *Plasmodium falciparum*, the highly polymorphic *var* genes encode the major virulence protein, PfEMP1, which bind a range of human receptors through varying combinations of DBL and CIDR domains. Here we present a tool, Varia, to predict near full-length gene sequences and domain compositions of query genes from database genes sharing short sequence tags. Varia generates output through two complementary pipelines. Varia_VIP returns all putative gene sequences and domain compositions of the query gene from any partial sequence provided, thereby enabling experimental validation of specific genes of interest and detailed assessment of their putative domain structure. Varia_GEM accommodates rapid profiling of *var* gene expression in complex patient samples from DBLα expression sequence tags (EST), by computing a sample overall transcript profile stratified by PfEMP1 domain types.

**Results:**

Varia_VIP was tested querying sequence tags from all DBL domain types using different search criteria. On average 92% of query tags had one or more 99% identical database hits, resulting in the full-length query gene sequence being identified (> 99% identical DNA > 80% of query gene) among the five most prominent database hits, for ~ 33% of the query genes. Optimized Varia_GEM settings allowed correct prediction of > 90% of domains placed among the four most N-terminal domains, including the DBLα domain, and > 70% of C-terminal domains. With this accuracy, N-terminal domains could be predicted for > 80% of queries, whereas prediction rates of C-terminal domains dropped with the distance from the DBLα from 70 to 40%.

**Conclusion:**

Prediction of *var* sequence and domain composition is possible from short sequence tags. Varia can be used to guide experimental validation of PfEMP1 sequences of interest and conduct high-throughput analysis of *var* type expression in patient samples.

**Supplementary Information:**

The online version contains supplementary material available at 10.1186/s12859-022-04573-6.

## Background

Pathogens can evade the immune system through polymorphic protein families interacting with host molecules. Examples of such gene families are the PIR and *var* in *Plasmodium*, the VSG in *Trypanosoma brucei* and the *srs* in *Toxoplasma* [1]. The most studied gene family is probably the *Plasmodium falciparum var* genes, which encode the *P. falciparum* membrane protein 1 (PfEMP1) family. PfEMP1 variants are inserted into the erythrocyte membrane to bind specific human endothelial receptors [2]. The PfEMP1 are targets of acquired immunity, and in response, the protein family has expanded and diversified to each parasite genome containing ~ 60 copies of PfEMP1-encoding *var* genes of 6–12 kb [3–5]. The extracellular part of PfEMP1 (encoded by the *var* exon1) is composed of multiple DBL (Duffy binding like) and CIDR domains capable of binding specific human receptors. DBL and CIDR domains are classified into a few main domain types, DBLα-ζ and CIDRα-γ, which have been further divided into domain subtypes [4]. Due to varying sequence diversity homogeneity between main domain types, the stringency by which domain subtypes are defined differs between main domain types. A comprehensive description of PfEMP1 domain classification can be found in [6, 7].

**Fig. 1:**
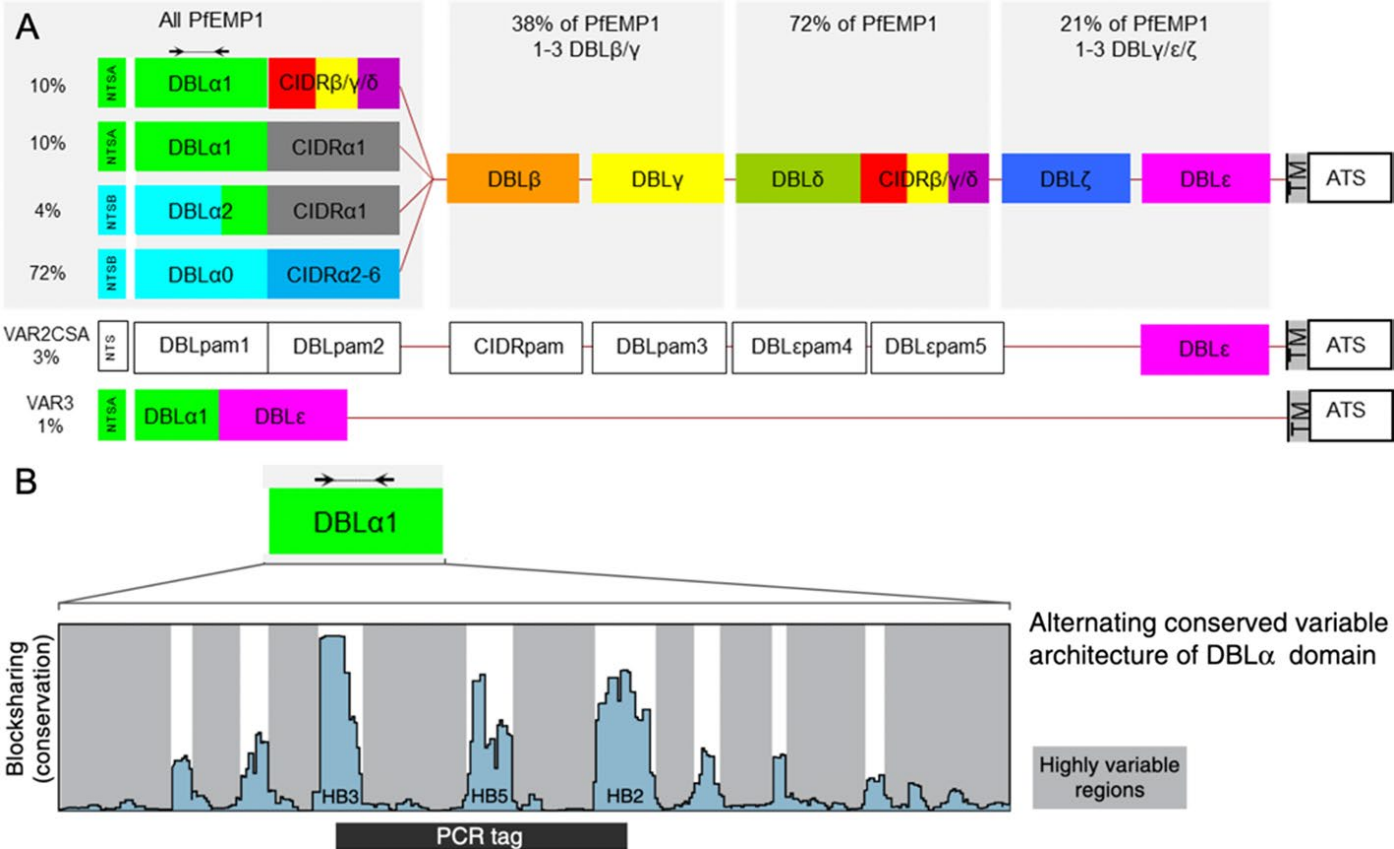
Overview of *var* gene-encoded PfEMP1 domain structures and the common distribution of different types and domain compositions of *var* genes in the average *P. falciparum* genome. **A** Arrows indicate location of commonly used annealing sites for degenerate primers, which can target the genes to generate DBLα sequence tags. Domains are coloured differently according to main domain type. DBLα2 domains are products of recombination between DBLα0 and DBLα1 domains. DBLα1 and DBLδ can have either a CIDRβ, CIDRγ or CIDRδ domain. **B** Shows block structure of DBLα domain (adapted from [8])

The domain composition of each PfEMP1 can vary but follows a general pattern (Fig. 1), which includes an N-terminal NTS-DBLα-CIDR “head structure” followed by varying combinations of domains in a semi-conserved order of main domain types. The exception to this rule is the highly conserved *var2csa* and *var3* genes, which encode atypical domain compositions. The anchoring intracellular part of PfEMP1 is encoded by the *var* exon2.

Characterization of novel *var* genes and analysis of *var* gene expression in patient samples is difficult due to the diversity of the genes. Although it is possible to obtain full-length *var* genes by assembling data from full genome sequencing, assembly and expression profiling from RNA sequencing is error prone, expensive and often challenging due to the small volumes of blood which can be drawn from severely ill children [9]. A cost-effective alternative has been to sequence reverse transcription (RT)-PCR-amplified DBLα ESTs, and use these to quantify the relative expression level of different *var* specimens in a patient sample, such as in [10–13]. Analyses of diversity of DBLα tags amplified from gDNA are also employed to study dynamics of parasite populations [14].

Until now it has not been possible to infer the domain composition of the entire encoded PfEMP1 from short sequence tags. However, as the DNA sequence diversity in the DBLα-tag region is extensive, it is possible that the flanking sequence of the originating genes can be predicted from the DBLα-tag, if sufficient information on global sequence diversity is available.

Recently, through whole genome sequencing of 2400 parasites collected across the world, a database of over 140,000 *var* genes was generated [6]. We sought to exploit this unprecedented sequence depth and extend it with a further 750 samples to build and test a tool, which will enable reconstruction and experimental validation of the near full-length variant genes from any sequence tag available. Specifically built for *var* genes, we extended the tool to also allow rapid quantification of PfEMP1 domain-specific expression in complex malaria patient samples by analysis of *var* DBLα expression tags.

### Implementation

**Fig. 2:**
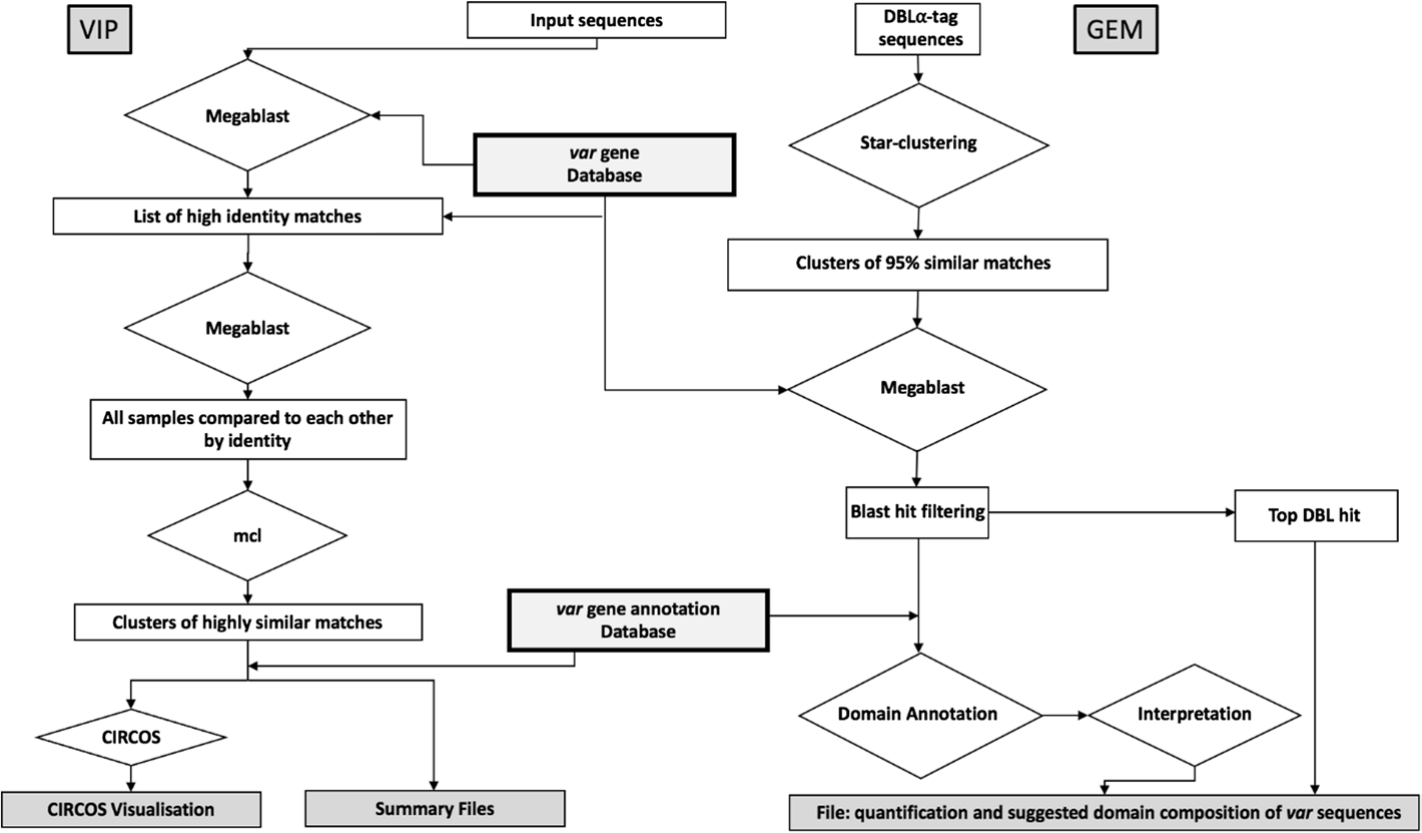
Flowchart of Varia pipelines. Filled rectangles indicate output files; diamonds indicate tools and data processing

Varia offers two analysis pipelines (Fig. 2). In the *Var* Identification and Prediction (Varia_VIP) pipeline, the user provides one or more partial *var* sequences, which are used for searching a *var* database for near identical sequences. Hit sequences are clustered based on their full-length sequence similarity, and the domain composition of the longest gene sequence representing each cluster is visualized in circular plots and tabular output files (Additional file 1: Figures S1-S3). Note that the VIP module can be used on any gene family, if a reference database is provided.

In the Varia Gene Expression Module (Varia_GEM), batches of DBLα ESTs, such as those generated by high-throughput sequencing of multiple patient samples [11], are clustered to show the distribution of unique DBLα-tag sequences in each data batch. For each unique DBLα-tag, near identical sequences in the *var* database are identified and their domain composition processed to generate a single consensus prediction of the domain composition of each query gene, as well as the relative expression level of all known PfEMP1 domains in each batch of sequences. The output is given in Excel file format (Additional file 1), allowing subsequent statistical analyses of domain type association with e.g. clinical data pertaining to the samples.

For Varia, a new *var* genome database containing all assembled *var* gene contigs and their domain annotations was generated. The database includes 2400 Illumina sequenced parasite isolates (https://www.malariagen.net/projects/Pf3k and https://www.malariagen.net/projects/p-falciparum-community-project) assembled in [6], 15 Pacific Bioscience sequenced *P. falciparum* clones [3, 6] as well as newly assembled *var* genes of 755 *P. falciparum* isolates from the Community Project. The *var* genes of the 755 isolates were assembled using the *var* gene assembly pipeline as described in [6] and the sequences were added to https://github.com/ThomasDOtto/varDB/tree/master/Datasets/Additional755/. Not all genes from clinical isolates are full-length *var* genes, and in general, the exon 2 is missing from many of these genes. As we are interested in the prediction of sequences and domains, sequences shorter than 3 kb were excluded. We merged the new dataset with the existing, resulting in a total of 205,595 *var* gene sequences. From translated amino acid sequences, domain subtypes described in [4] were annotated using HMMer models developed in [6]. We used the command “hmmscan –cpu 12 –noali -E 1e-6 –domE 1e-6” and parsed the results with an in-house Perl script. The Perl script and the HMMer models for the prediction are available at https://github.com/ThomasDOtto/varDB/. As annotation was performed on complete and incomplete *var* sequences, these criteria may leave some sequence ends with incomplete domains unannotated.

**Fig. 3:**
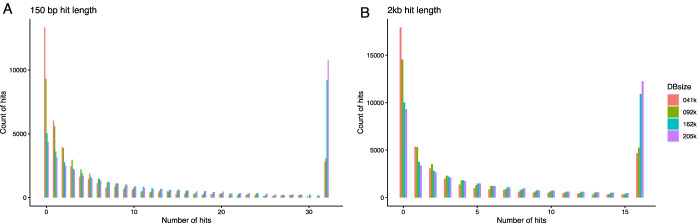
Overview of database hits to 40 k test sequences in databases of different size. **A** Distribution of the number of database hits to 40 k 150 bp sequences (all starting from LARSFADIG in DBLα) in the four databases. (Domains with ≥ 32 hits are summed under 32). **B** Distribution of 2 kb query genes by number of hits in databases of different size (Domains with ≥ 16 hits are summed under 16)

To explore the *var* sequence prediction from this database, we randomly selected 40 k DBLα sequences from the database. Specifically, we extracted the first 150 nucleotide bases starting from the LARSFADIG motif found in all DBLα domains. These sequences were blasted (megablast version 2.2.26, parameters: “-F F, E-value cutoff 1e-3”) against four different partitions of the database: (1) the newly generated *var* sequences for this paper (41 k sequences), (2) the “normalised dataset” defined in [6] as the global representation of *var* gene diversity (720 genomes, 81 sequences, 92 k sequences) (3) the full dataset analysed in [6] (2400 isolates, 162 k sequences, labelled as Version 3) and 4) all the full compiled database (205,595 sequences; considered database version 4). A database sequence was considered a hit if it matched the DBLα query sequence with at least 99% identity over a 150 base pair overlap (self-hits were excluded). In addition to this, we analysed database hits using the full-length gene sequence of the same 40 k query genes using megablast, as described. We counted database hits matching (> 99% identity) the first 1000, 2000 and 3000 base pairs following the LARSFADIG motif and hits matching > 80% of the length of the complete input sequence (Table 1 and Fig. 3). The two query files can be found in the GitHub repository within the SimilarityTests directory.

**Table 1:** Proportion of 40 k *var* sequences of different length with identical sequence hits (> 99% identity) against databases of different size

Hit length	Databases			
	41 k (%)	92 k (%)	162 k (%)	205 k (%)
150 bp	67.1	77.0	87.6	89.3
150 bp (Africa)	58.9	70.2	83.2	85.4
150 bp (Asia)	84.8	91.7	96.6	97.4
1 kb	58.5	68.1	79.9	81.8
2 kb	55.7	64.1	75.3	77.0
3 kb	46.5	53.8	63.3	64.7
full hits (80%)	30.6	41.8	53.4	55.7

All sequences start at the LARSFADIG motif found in the N-terminal DBL domain (DBLα) of most *var* genes. Query sequences from African and Asian genes are shown separately for the 150 bp sequences

## Results

First, we evaluated the opportunities we can achieve with Varia. As expected, increasing the database size resulted in an increased proportion of query genes with database matches and more hits per query gene. However, even with the largest database, more than 10% of 150 bp query sequences are without any hit. The *var* gene database is biased towards genomes from South East Asian parasite isolates [6], and larger diversity has been reported among *var* genes of African isolates compared to Asian isolates. Separating the 40 k 150 bp query sequences by African or Asian origin showed that Asian sequences were more likely to have a database match (97%) than African sequences (85%). The hit rate drops with the length of query sequence, and for full-length sequences just over 50% of the sequences have complete matching hits. Increasing the database size from 160 to 220 k makes little difference, but larger databases increase the hit frequency (Fig. 3). The most marked drop is seen when changing hit lengths from 2 to 3 kb, which resulted in hit rates going from ~ 77% to ~ 65%. This can be explained by the recombinogenic nature of *var* genes and the presence of a major recombination hotspot identified mid-*var* at 2–3 kb following the N-terminal DBLα-CIDR domain complex encoded in most PfEMP1 [4].

In practice, *var* tags are likely to be generated from sequencing of PCR-amplified fragments spanning across variable regions roughly corresponding to the second structural subdomain of DBL domains [4]. The varying sequence diversity homogeneity between main types of DBL domains is likely to affect the ability of Varia_VIP to predict correct domain compositions. To assess this, Varia_VIP predictions were made from DBL tag sequences corresponding to the second structural subdomain [4] of all main DBL domains found in *var* genes from the 15 long-read sequenced and annotated *P. falciparum* genomes. A total of 971 DBLα-ζ domain tags were run through Varia_VIP, excluding the test sequences from the database (Table 2).

**Table 2:** Analysis of database hits and gene predictions from Varia_VIP analysis of DBLα-ζ tags. Sample tags from 971 different DBLα-ζ domains were extracted from 15 *P. falciparum* genomes

Domain	No. tags tested	Hit rate (%)			Average No. of clusters			Percentage correctly annotated genes (any cluster)			Percentage correctly annotated genes (top 5 Clusters)			Percentage perfect DNA sequence hits (top 5 clusters)		
Seq. ID threshold →		99%	95%	90%	99%	95%	90%	99%	95%	90%	99%	95%	90%	99%	95%	90%
DBLα	293	95	99	100	14	34	92	72	75	78	66	62	53	29	27	22
DBLβ	127	94	96	97	13	41	100	73	73	73	69	65	53	39	35	27
DBLδ	256	96	99	100	15	34	60	74	74	77	69	63	55	33	29	26
DBLγ	138	91	93	93	18	61	146	71	70	71	64	55	44	41	36	24
DBLε	109	78	78	78	21	61	120	54	56	56	51	44	39	25	17	13
DBLζ	48	96	100	100	29	99	263	60	63	63	54	38	29	35	21	10

Tags were run through Varia_VIP using a length filter of 150 base pairs and an identity filter of 99%, 95% and 90%. The hit rate shows the proportion of queried tags that had one or more hits in the *var* gene database. The average number of clusters into which hit genes were grouped and the proportion of genes for which a correct domain subtype annotation was found in any cluster, or in the five clusters with most hit sequences (top 5), is shown. Also shown is the proportion of genes for which a sequence matching the reference gene by 99% identity over at least 80% of the full sequence was found among the top five Varia_VIP clusters

This showed that tags from DBLα, β, δ and γ domains resulted in fewer different hit sequences (grouped into clusters of identical hit sequences) and more frequent correct annotations compared to DBLε and DBLζ domains. This observation can be explained by the differences in the distribution of diversity within these main domain types. DBLα, β, δ and γ domains are highly diverse in sequence and diversity is homogeneous. Conversely, DBLε and DBLζ domain sequences distribute into distinctly different groups of highly similar sequences. Thus, as identical DBLε and DBLζ domains are found in many different PfEMP1 variants, correct predictions are difficult to make from these domain tags.

As expected, lowering the similarity threshold increased the proportion of queries with correctly predicted annotations but at the cost of a higher number of different predictions. Specifically, for the DBLα domain tags, which are found in the 5′-end of all *var* genes, a correct annotation was identified for 72% of the query genes at 99% sequence similarity threshold. The correct annotation was found among 14 suggested predictions, on average. At this 99% similarity threshold, prediction of the exact query DNA sequence, defined as 99% identity over at least 80% of the sequence, was successful for 29% of the DBLα domain tags. Lowering the similarity threshold to 90% resulted in a limited increase to 78% of the query genes with correct annotation among the suggested domain compositions, and an increase in the number of alternative suggestions. For this reason, we recommend applying the highest similarity threshold resulting in database hits when using Varia_VIP to search for putative domain compositions and sequences of query genes, to allow manageable experimental validation (e.g. by PCR) of the query sequence.

As noted above, the current *var* gene database is biased towards genomes from South East Asian parasite isolates [6]. At 95% sequence similarity over 200 base pairs, 98% of DBLα tags from African isolates had one or more database hits, whereas this was the case for 100% of DBLα tags from Asian isolates. This resulted in a correctly annotated cluster-representative sequence by Varia_VIP (any cluster) for 60% versus 85% of the query tags from African vs. Asian isolates, respectively.

In some studies, for example of *var* gene expression in patients, a rapid prediction of the single most likely PfEMP1 domain composition of a large number of sequences is required. For this, we built Varia_GEM, which, based on all hit sequences to a DBLα EST, returns the likely consensus domain annotation of the query gene. As the ability to predict correct annotations depends on domain type and distance to the query tag, Varia_GEM was designed to assess domain annotation for each domain position relative to the PfEMP1 N-terminal. For each domain position (D1–D10; DBLα is always at position D2) the tool determines if a specific PfEMP1 main domain type or domain subtype is dominant (> 66%) among hit gene sequences. If this is the case, the tool returns the consensus annotation in a tabular format, along with quantitative data of frequency of ESTs associated with the domain or domain composition, to allow quantitative analysis of PfEMP1 domain traits with e.g. clinical features pertaining to the origin of the sequences.

**Fig. 4:**
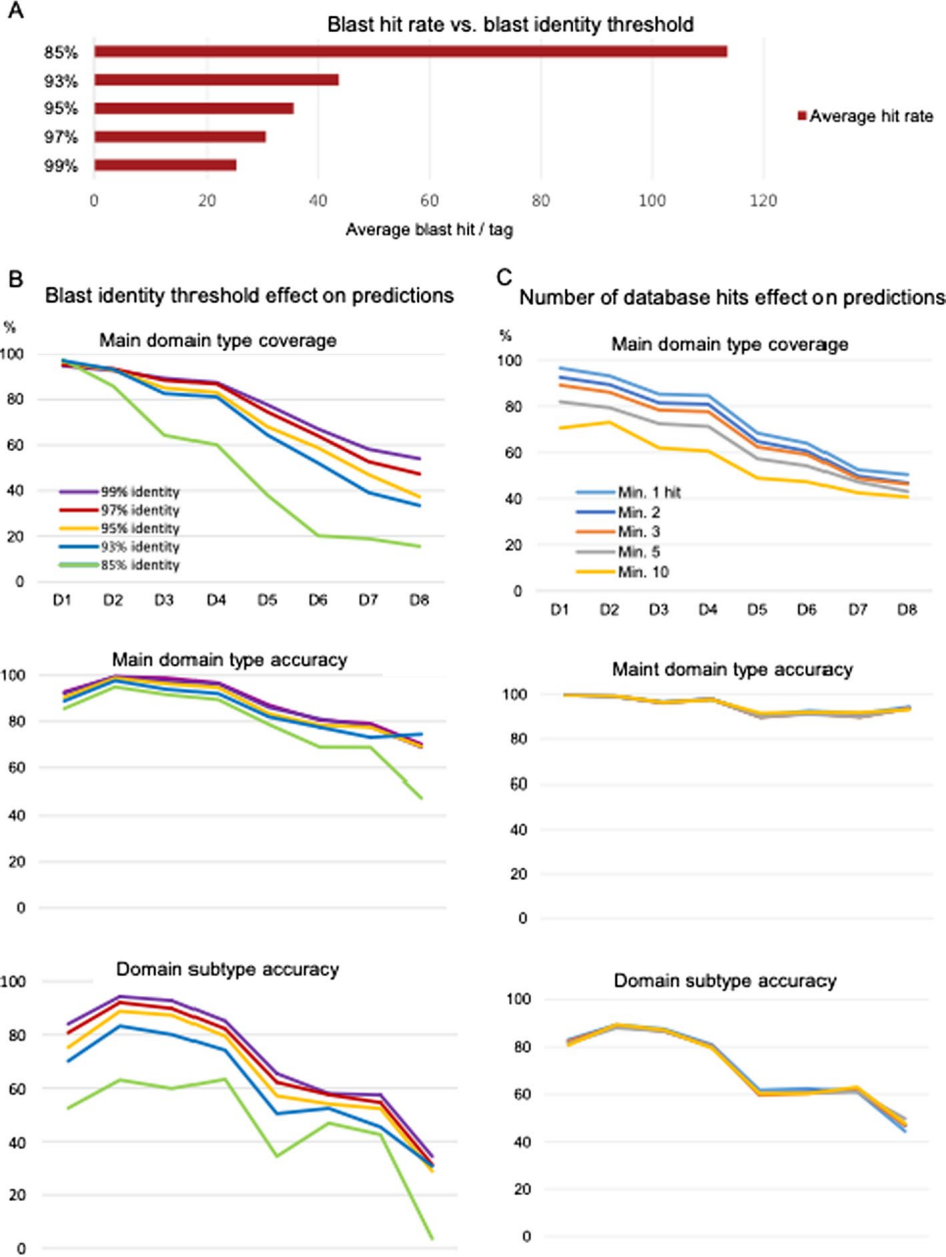
Analysis of database hits and gene predictions by Varia_GEM analysis of DBLα tags.** A** Average blast hit per query DBLα tag sequence at different blast identity thresholds. **B** Plots showing main domain type coverage and domain type accuracy of predictions across the first eight domain positions (D1–D8) for 660 DBLα tags extracted from genes encoding 220 DBLα0, 1 and 2 domains. Data shown for different blast identity thresholds (99–85%). **C** Domain prediction coverage and accuracy of predictions made at 95% blast identity threshold shown by number of database hits

To determine parameters for optimal domain type prediction by Varia_GEM, we tested main domain type and domain subtype predictions at different identity threshold values. We extracted 220 randomly selected DBLα0, 1 and 2 tag sequences (660 in total) from the *var* gene database, as they would be amplified by the commonly used DBLα EST primers [15]. The cognate genes were removed from the database, and the database was searched for genes sharing 99, 97, 95, 93% and 85% identity across 200 base pairs of the DBLα-tags. This resulted in an average ~ 25 hits per DBLα-tag at 99% threshold, increasing to 44 hits at 93% and 114 hits at 85% (Fig. 4A). The consensus domain composition generated for each DBLα-tag was compared to the known domain composition of the gene to calculate the accuracy of the predictions at each position of the domain (Fig. 4B). The accuracy was calculated both at the main domain type and domain subtype level, as follows:

- *Average main domain accuracy*: An annotation was considered incorrect if the predicted main domain type was wrong, or if a non-existing domain was predicted (i.e. a domain was predicted at a position where no domain was present in the original sequence).
- *Average domain subtype accuracy*: An annotation was considered incorrect if the predicted domain subtype was wrong, or if a non-existing domain was predicted (i.e. a domain was predicted at a position where no domain was present in the original sequence).

Next, the frequency by which the tool could predict the correct *var* gene domain compositions (coverage) was calculated for each domain position.

- *Average coverage (main domain type):* The proportion of genes with a correctly predicted main domain type at said position. A domain annotation was considered incorrect if a wrong main domain type was predicted, or a domain annotation was missing (i.e. no domain type was predicted where a domain was present in the original sequence).

Lowering the blast identity threshold and thereby including a higher number of hits resulted in fewer predictions and fewer correct domain annotations (Fig. 4B). However, main domain predictions were robust, with 85% accuracy over the first four domains. Lowering the blast identity threshold from 99 to 93% caused a slight decrease in main type accuracy, but a moderate decrease in coverage and domain subtype accuracy. Lowering the identity threshold further to 85% caused a significant drop in main domain coverage and domain subtype accuracy, but again only a small decrease in main domain type accuracy. The most significant effects from lowering the blast identity threshold, was seen on domains at position D5-8. The level of coverage and accuracy was similar for different subtypes of DBLα (DBLα0, 1 and 2, not shown).

Most frequent and correct domain predictions were generated using the 99% blast identity threshold. This threshold is likely to be too stringent for analysis of sequencing data, which may contain various minor sequence errors. Instead, a 95% threshold was chosen as default for Varia_GEM. This threshold results in a 0–10% drop in main domain type coverage and domain subtype accuracy, as well as a 0–3% decrease in main domain type accuracy, on average. Using the 95% identity threshold, the effect of the number of blast hits on domain prediction was investigated (Fig. 4C). This showed that a higher number of hits adversely affected the coverage but did not affect the accuracy of the predictions, and that including predictions made based on only one hit sequence resulted in most frequent correct predictions. Based on this, Varia_GEM was set to predict on every tag regardless of the number of database hits.

## Discussion

Prediction of variant genes from short sequence tags is challenged by the speed and molecular mechanisms by which the genes evolve. Here, we empirically explored to which degree we can predict *P. falciparum var* genes from short DNA sequence tags. Previous studies have shown that the extensive diversity of the *var* genes is ancient and in large depends on recombination [6]. This challenges prediction of *var* genes. Moreover, prediction is hampered by an ambiguous clustering of PfEMP1 domains into specific subtypes. Main domain types (DBLα-ζ, and CIDRα-γ) are well defined (Figs. 3B, 4), but the sequence diversity homogeneity differs between main domain types, and domain subtyping is uncertain [4, 6] and possibly reflects antigenic rather than functional diversification [18]. For example, found at domain position 3, CIDRα domain sequences separate distinctly into two groups, CIDRα1 or CIDRα2-6 domains, but the 18 subsets of CIDRα2-6 domains, all expected to bind CD36, are less well segregated and defined. The recombinogenic nature of *var* genes makes it likely that gene and domain predictions will be most accurate nearest to the analyzed tag and most uncertain across sites of frequent recombination. This explains the drop in main domain coverage and domain subtype accuracy with domain position, and the significant drop in correct predictions around domain position D5 (Table 1), which corresponds to a major recombination hotspot found mid-*var* gene [4]. However, the high overall main domain accuracy of Varia predictions across all domain positions shows that if a main domain type is predicted, this is very likely to be true.

**Fig. 5:**
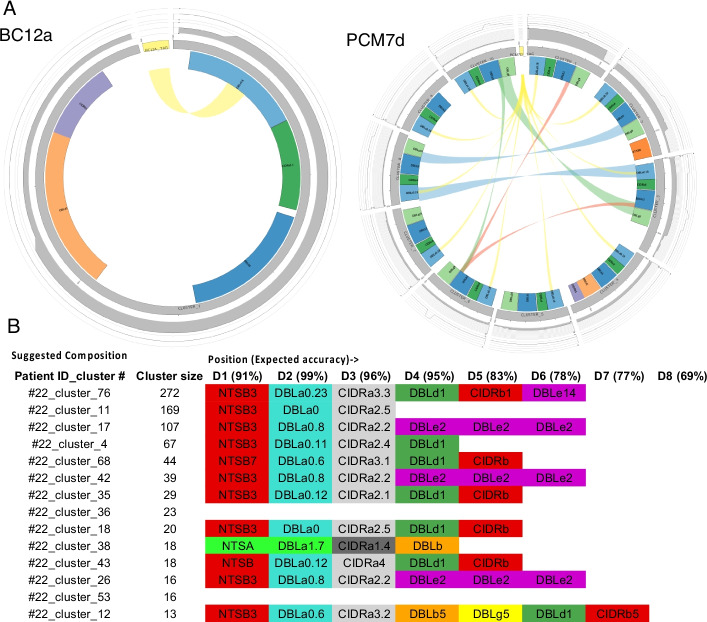
Examples of Varia use and output. **A** Circos plot of domain-annotated full-length *var* genes suggested by Varia_VIP to represent the full length sequence from a DBLα-EST analysis of [16]. The BC12a tag returns one and the PCMd7 tag returns nine different sequence clusters. The format of plots is detailed in the Additional file 1, but in short, each segment represents a sequence cluster from a single patient. The coloured boxes are the domain annotations, and the coloured ribbons between domains are blast hits between the suggested sequences (the colour depends on the colour of the domain. The yellow box is the input sequence). For each cluster, the blast hit to other gene clusters (outer graph, correlates with blast ribbons) and the number of hits within the cluster (bar plot) is shown. **B** Section of the Varia_GEM output file generated from DBLα ESTs amplified from patient number 22 in a recent study of P. falciparum infected travelers [17]. Identical DBLα_ESTs are clustered, and the read counts represent the relative expression level of each specific DBLα EST tag. Here, DBLα EST sequence cluster number 76 was the most prominently expressed gene. The Varia_GEM-predicted domain composition of the full-length transcripts is shown for each DBLα EST. The output file also includes these data stratified per domain type such that the overall relative expression level of e.g. CIDRα1 domains can be assessed for each single patient and all patients in a dataset. The expected accuracy of main domain type prediction at each domain position based on the current database is shown

As an example of Varia_VIP use, we predicted the full *var* gene sequences from DBLα tags PCR-amplified in [16]. In this study, the authors experimentally validated five full-length *var* genes from the tags. Figure 5A shows the different full-length genes Varia suggests are the origin of a PCR-amplified DBLα tag for two of the DBLα tags (Complete results in Additional file 1: Table S1). The Circos plot visualizes the options to design primers to experimentally verify the *var* gene from a few potential sequences. Overall, Varia found the full sequences in the first two clusters, see Additional file 1. As an example of Varia_GEM use, Fig. 5B shows a section of the output file of the predicted *var* gene expression profile of one of the 32 *P. falciparum*-infected adult travellers returning to Germany [17]. A comprehensive comparison of the RNA-seq and DBLα-EST and Varia_GEM predicted *var* expression profiles are given in [17]. In brief, a partial domain annotation was made for ~ 85% of DBLα-ESTs; ~ 83% of all unique DBLα-ESTs were found in the RNA-seq approach, and 82% of the most abundant transcripts encoding a DBLα-tag region (upper 75th percentile of RNA-seq contigs) were also found by the DBLα-tag approach.

## Conclusions

Due to the diversity of PfEMP1 sequences, resolution of unknown full-length *var* gene sequences and their domain structure has required cumbersome laboratory proceedings. With Varia, we generated a tool that predicts *var* gene sequences from small easily obtained sequence fragments, to allow the community to reconstruct genes of interest. The likelihood of correct predictions of sequence and domains flanking the query tags was high, whereas predictions distant to the query tag were less likely. These limitations are caused by the sequence diversity and recombination history of the *var* genes. Improved rates of prediction may be achieved if additional *P. falciparum* genomes, in particular from Africa or South America, are added to the *var* gene database. However, with awareness of these limitations, sequences and domain compositions of *var* genes of interest can be experimentally validated by PCR, and differential *var* type expression between groups of patient samples can be assessed. The Varia tool will thus be useful to understand the distribution and clinical importance of different *var* gene subsets, and can be adapted to predict sequence and domain compositions from tags of other variable gene families.

## Availability and requirements

**Project name:** VARIA,

**Project home page:** https://github.com/GCJMackenzie/Varia,

**Operating system(s):** Linux (Possible to use through Virtual box for Max and Windows),

**Programming language:** Bash and Python,

**Other requirements:** Database can be downloaded from https://github.com/ThomasDOtto/varDB**,** as indicated on VARIA GitHub page. Varia builds on several basic bioinformatics tools, like NCBI BLAST, mcl, Circos and samtools. **License:** GNU General Public License v3.0,

**Any restrictions to use by non-academics:** no restrictions.

## Supplementary Information

**Additional file 1**: Information on implementation and usage of Varia. Examples of output.

## Data Availability

All data can be found at https://github.com/GCJMackenzie/Varia. The database with the var gene sequences can be found at https://github.com/ThomasDOtto/varDB.

## Abbreviations

EST: Expression sequence tags
CIDR: Cysteine‐rich inter domain region
DBL: Duffy binding-like
PfEMP1: *P. falciparum* Membrane protein 1
