# Supplementary material for: Varia: a tool for prediction, analysis and visualisation of variable genes
Source: BMC Bioinformatics. 2022 Jan 24;23:52. doi: 10.1186/s12859-022-04573-6 (PMC8785495; doi:10.1186/s12859-022-04573-6)
Supplement: Supplementary file 1 — Additional file 1: Information on implementation and usage of Varia. Examples of output. [file 12859_2022_4573_MOESM1_ESM.pdf]

Supplementary data for
Varia: Prediction, analysis and visualisation of variable genes

Additional implementation description of Varia pipelines
Varia has two pipelines: The *var* identification and prediction (Varia_VIP) and the Gene Expression Module (Varia_GEM).

***Var* Identification and Prediction (Varia_VIP) pipeline**
Arguments
Varia_VIP is run using the following command line:

Varia.sh VIP [optional arguments] -i [input tag file]

-i is the only mandatory argument required to run Varia_VIP as this specifies the input file to be used. Varia_VIP also has a number of optional arguments, which can be used to change the output directory and change various filters used throughout the module, a detailed list of these options and their default settings can be found in the readme file, or by using:

Varia.sh VIP -h

Database choice
The database used by Varia_VIP is selected as part of the installation script. As default the program is set to use the provided paired "*var* sequence" and "*var* annotation" databases. Once these databases are downloaded, execution of the installation script, Install_Varia.sh, will prepare the database files for downstream analysis (generating the search indexes). The default database should be downloaded from https://github.com/ThomasDOtto/varDB/tree/master/Datasets/Varia/.

The *var* sequence and *var* annotation database files will be soft-linked into the Varia domains subdirectory. This enables the user to use other databases (e.g. to apply Varia to other variable gene families). To do this, the sequence database should be in fasta file format, and an accompanying domain annotation file containing matching entries should be formatted using formatdb from the NCBI blastall package. The user can change databases used by Varia by deleting soft links to the fasta index and database files, then rerunning the installation script.

Environment setup and input data
Input sequence tags should be provided to Varia_VIP in fasta file format. A batch of multiple fasta-formatted sequence tags can be submitted in a single input file and Varia_VIP will run each tag individually. Each tag must be longer than 200 bps (default setting) as this minimum length is later used to filter the database blast results.

As input parameters, Varia_VIP requires the name of the input tag file. In addition, the user can modify several parameters, such as the *identity cut-off* used for filtering database hits. After checking the validity of parameters given, a new result directory [name of input file]-[identity filter]-Varia_Out is generated (name and location can be changed with the -o parameter).

Search for tag-related sequences in the var sequence database

After the environment is set up, the input file is duplicated into a temporary file to allow for modifications without altering the original file. The first input tag is extracted from the input file, placed in a temporary file and compared to the sequence database using Megablast [1]. If there are no database hits, VARIA_VIP will move onto the next input tag. For each tag with a database hit, the output blast file is filtered to collect the names of all database hits that are longer than the "database length filter" (-l parameter, default 200 bps) and higher than the "database identity filter" (-f parameter, default 99%).

Comparison and clustering of hit sequences

Next, the names of the filtered hits and the *samtools faidx* tool are used to create a new fasta file containing the sequence for each hit. *Formatdb* is used to temporarily make this fasta file into a database that Megablast can use in a blast-all-against-all search to generate an output file containing every region of alignment between each gene and every other gene in the fasta file. These results are filtered to include all results with sequence identities higher than the "self-blast identity filter" (-c parameter, default 99%) over the percentage of length set by the "self-blast length filter" (-p parameter, default 80% of either the aligned sequences length). The sequence name and alignment length are extracted from each filtered hit and formatted for Markov Clustering (*mcl*) of the sequences into clusters of near identical sequences. Due to high sequence variation between *var* genes, it is recommended to use the default self-blast settings of 99% identity and >80% length of either sequence criteria to ensure members of a cluster are highly similar both in sequence and domain composition. The largest sequence in each cluster is then used as a representative of the cluster, for example in the circus plots. Clusters are numbered according to number of hit sequences included, i.e. cluster 1 contains most hits.

Plot generation

The representative sequence of all clusters relating to a specific query tag, and their domain composition extracted from the annotation database, are then formatted for visualization in *Circos* plots. *Circos* allows multiple plots to be shown for each cluster. Annotation pertaining to each sequence is presented around the circumference of the visualisation. Figure S1 shows different types of information annotated to an individual sequence in different tracks (A-E) radiating out from the centre of the entire visualisation. Varia provides several input files for track labelling and other features. The display of the different features is stored in the "Varia.conf" *configuration file,* see below.

All input files needed to generate the plots are retained by Varia to allow users to customise the plot of a single sample.

Configuration File: Varia_VIP uses a standard configuration file for all *Circos* plots. This file details what tracks are present, the input files to be used for them, the positioning of tracks and labels and formatting of text size and font etc. Given the large number of parameters, it is recommended that users edit a copy of the provided Varia configuration file if should they wish to alter the plot. Directly editing the configuration file will alter all plots Varia makes from then on. The configuration file "Varia.conf" is found in the "Varia1_5/scripts/" directory.

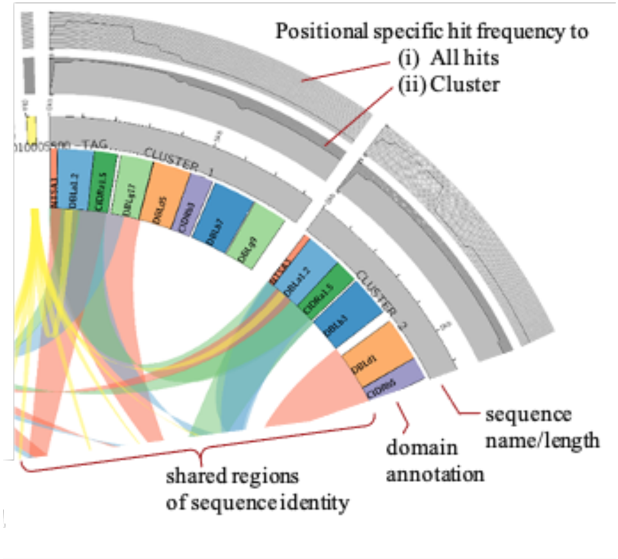

Figure S1. Examples of Varia output and performance. Section of a Circos plot from Varia_VIP showing sequence similarities and domain traits of database genes similar to a query DBLα tag (yellow).

Cluster Track: Grey track labelled "sequence name" on Figure S1. The cluster or "chromosome track" is the only mandatory track in a *Circos* plot. Each cluster-representative sequence is labelled by its cluster number and acts as an x-axis for other annotation plots relating to the cluster. Ticks represent 1000 bp intervals.

Domain annotation track: This track shows the encoded PfEMP1 domain composition of the cluster-representative sequence. Each block on the plot represents a domain and shows its size and positioning along the length of the largest sequence. These blocks are labelled based on the sequence annotation database selected during installation, the soft link to the file *vardb_domains.txt,* which can be found in the domains directory.

The colour of the blocks is determined based on the domain name and what colour is assigned to it in the colour map file *domain_color_map.txt* in the "domains" directory. The user can change the colours by editing this file.

Ribbon track: Labelled "shared regions of sequence similarity" Figure S1. The ribbon plot is used to show regions that share at least 200bp and an identity ≥ 99% between the largest sequences of other clusters. Each ribbon will show the position and length of regions that are nearly identical between two cluster-representative sequences. The colour of links cycles between red, blue and green to help differentiate the ribbons on a plot and show overlapping ribbons matching to the same place. Ribbons between the input tag and other clusters, are shown in yellow. There should always be a link between the input tag and every other cluster.

Inter-cluster coverage tracks: Most outer track on Figure S1. This plot is a quantification of the information shown on the ribbon plot. Here, the Y axis shows the number of plotted sequences that share identical across a region of 100 base pairs. To obtain this, an array of integers is set up for

each base pair position in the sequence. For each position, the ribbon plot file is searched for matches to the plotted sequence, and when a match is found, 1 is added to each base pair position within the range of the ribbon. Once the ribbon plot file has been fully searched, the array is split into 100bp segments and the median is calculated for that segment. The largest coverage value for the entire plot is then set as the maximum Y value for the entire plot. Finally, files are generated to display the Y axis and the maximum and minimum values.

Intra-cluster coverage track: Second outer track on Figure S1. Similar to the inter-cluster coverage plot, this plot shows the regions of matching sequence shared between genes contained within the cluster. The genes in each cluster are compared to the largest gene in that cluster and the results are mapped into an array as detailed above and the median is found for each 100 bp segment. Max value is found, and files are generated to show the Y axis.

Summary Files
For each input sequence tag, summaries of the extracted database hit sequences are also returned in excel-readable tables.

Cluster summary (Figure S2)**:** The *cluster summary* file shows information for every gene that was clustered by mcl, as a separate entry. Each entry shows the cluster number and name, the name of the gene, the length of the gene in base pairs, the country where the gene was isolated and the encoded domain composition. If specific information points are lacking, "n/a" is reported. An example can be found on the GitHub site, in the example directory.

Final summary (Figure S3)**:** The *final summary* file shows information on each cluster-representative sequence returned by Varia_VIP. Each entry in the file shows: cluster name, the number of genes in the cluster, the name, sequence length and encoded domain composition of the longest gene (the cluster-representative sequence) in the cluster and, the number of different countries reported in the cluster. An example can be found on the git-hub, under Example/VIP/output.

Cluster_ID	Seq_ID	Seq_length	Country_Distribution	Subdomains
1	PH0444-C.g34	5035	Cambodia	NTSA3 DBLa1.2 CIDRa1.5 DBLg17 DBLd5
1	PH0194-C.g145	8319	Cambodia	NTSA3 DBLa1.2 CIDRa1.5 DBLg17 DBLd5 CIDRb3 DBLb7 DBLg9
1	PH0241-C.g416	8262	Cambodia	NTSA3 DBLa1.2 CIDRa1.5 DBLg17 DBLd5 CIDRb3 DBLb7 DBLg9
1	PH0820-C.g184	8262	Cambodia	NTSA3 DBLa1.2 CIDRa1.5 DBLg17 DBLd5 CIDRb3 DBLb7 DBLg9
1	PH0823-C.g23	8319	Cambodia	NTSA3 DBLa1.2 CIDRa1.5 DBLg17 DBLd5 CIDRb3 DBLb7 DBLg9
1	PH0824-C.g47	8262	Cambodia	NTSA3 DBLa1.2 CIDRa1.5 DBLg17 DBLd5 CIDRb3 DBLb7 DBLg9
1	PH0839-C.g640	8262	Cambodia	NTSA3 DBLa1.2 CIDRa1.5 DBLg17 DBLd5 CIDRb3 DBLb7 DBLg9
1	PC0016-C.g67	8262	Kenya	NTSA3 DBLa1.2 CIDRa1.5 DBLg17 DBLd5 CIDRb3 DBLb7 DBLg9
1	PH0152-CW.g96	8214	Cambodia	NTSA3 DBLa1.2 CIDRa1.5 DBLg17 DBLd5 DBLb7 DBLg9
1	PH0390-C.g5129	8262	Cambodia	NTSA3 DBLa1.2 CIDRa1.5 DBLg17 DBLd5 CIDRb3 DBLb7 DBLg9
1	PH0819-C.g583	3147	Cambodia	NTSA3 DBLa1.2 CIDRa1.5 DBLg17
1	PD0863-C.g901	7077	Thailand	NTSA3 DBLa1.2 CIDRa1.5 DBLg17 DBLd5 CIDRb4 DBLb9
1	PH0291-C.g8	8147	Cambodia	CIDRa1.5 DBLg17 DBLd1 CIDRb3 DBLb7 DBLg9
2	PV0303-C.g485	6023	Vietnam	NTSA3 DBLa1.2 CIDRa1.5 DBLb3 DBLd1 CIDRb5
2	PH0332-C.g290	6444	Cambodia	NTSA3 DBLa1.2 CIDRa1.5 DBLb3 DBLd1 CIDRb5
2	PH0545-C.g745	6444	Cambodia	NTSA3 DBLa1.2 CIDRa1.5 DBLb3 DBLd1 CIDRb5
2	PH0555-C.g426	6444	Cambodia	NTSA3 DBLa1.2 CIDRa1.5 DBLb3 DBLd1 CIDRb5
2	PH0556-C.g464	6444	Cambodia	NTSA3 DBLa1.2 CIDRa1.5 DBLb3 DBLd1 CIDRb5
2	PH0557-C.g457	6444	Cambodia	NTSA3 DBLa1.2 CIDRa1.5 DBLb3 DBLd1 CIDRb5
2	PH0574-C.g356	6444	Cambodia	NTSA3 DBLa1.2 CIDRa1.5 DBLb3 DBLd1 CIDRb5
2	PH0717-C.g208	6444	Cambodia	NTSA3 DBLa1.2 CIDRa1.5 DBLb3 DBLd1 CIDRb5
2	PD0476-C.g308	6501	Thailand	NTSA3 DBLa1.2 CIDRa1.5 DBLb3 DBLd1 CIDRb5
2	PD0492-C.g677	6444	Thailand	NTSA3 DBLa1.2 CIDRa1.5 DBLb3 DBLd1 CIDRb5

Figure S2 Example of Varia_VIP output showing the beginning of the cluster summary file for input tag PfDd2_010005500:532-814. Columns from left to right: The cluster number each entry is assigned to; the sequence ID of the Varia database hit sequence; the length in base pairs of the hit sequence; the sequence country of origin; and the domain subtype composition of the hit gene.

Cluster_name	Cluster_size	Longest_seq	length	80%_matches	Coutry_distrib	Subdomains
Cluster 1	13	PH0194-C.g145	8319	7	3	NTSA3 DBLa1.2 CIDRa1.5 DBLg17 DBLd5 CIDRb3 DBLb7 DBLg9
Cluster 2	11	PD0476-C.g308	6501	10	3	NTSA3 DBLa1.2 CIDRa1.5 DBLb3 DBLd1 CIDRb5
Cluster 3	7	PD0872-C.g203	6813	6	1	NTSA3 DBLa1.2 CIDRa1.5 DBLg11 DBLd1 CIDRb5
Cluster 4	3	PV0113-C.g13	10608	0	2	NTSA3 DBLa1.2 CIDRa1.5 DBLg17 DBLd5 CIDRb3 DBLb7 DBLg9 DBLd1 CIDRb1
Cluster 5	2	PH0151-CW.g22	8229	1	1	NTSA3 DBLa1.2 CIDRa1.5 DBLg7 DBLd5 CIDRb3 DBLb9 DBLg9
Cluster 6	2	PD0512-C.g297	7665	0	1	NTSA3 DBLa1.2 CIDRa1.5 DBLb3 DBLg11 DBLd1 CIDRb6
Cluster 7	2	QG0236-C.g2368	6885	1	1	NTSA3 DBLa1.2 CIDRa1.5 DBLb3 DBLd1 CIDRb1
Cluster 8	1	PT0257-Cx.g2301	8274	0	1	NTSA3 DBLa1.2 CIDRa1.5 DBLg17 DBLd5 CIDRb3 DBLb6 DBLg11
Cluster 9	1	PF0819-C.g2715	7742	0	1	NTSA3 DBLa1.2 CIDRa1.5 DBLg17 DBLd5 CIDRb3 DBLb6
Cluster 10	1	PH0141-CW.g1	8961	0	1	DBLa0.3 CIDRa1.5 DBLg11 DBLd5 CIDRb4 DBLe11 DBLz2

Figure S3 Example of Varia_VIP output showing the final summary file for input tag PfDd2_010005500:532-814. Columns from left to right: The cluster number; the number of sequences in the cluster; the sequence ID of the longest database hit sequence in the cluster i.e. the cluster-representative sequence; the length in base pairs of the longest hit sequence; number of sequences in the cluster that are 99% identical over at least 80% of the length to the largest sequence in the cluster; the number of different countries of origin in the cluster; the domain subtype composition of the cluster-representative sequence.

Gene Expression Module (Varia_GEM) pipeline

Environment setup and input data

One or more input FASTA files containing DBLα tag sequences are required for Varia_GEM to run. The parameters are similar to the VIP module with the addition of -c - *min_cluster_size*. If specified

in the command line, the program will exclude downstream analyses of sequences representing clusters containing less than the *min_cluster_size* number of sequences. Default *min_cluster_size* setting is 10 sequences per cluster.

The program creates temporary fasta files, where each sequence is written and for each fasta file, Varia_GEM creates a subfolder for the output files generated downstream.

Clustering of sequences and search for tag-related sequences in the var sequence database
For each fasta file, DBLα tag sequences are clustered into groups sharing at least 95% nucleotide identity across minimum 200 bps using *Vsearch[2]* and written to separate cluster files in the fasta file subfolder, numbered from 1 to n. One sequence from each cluster is then used to search for genes with similar DBLα tag sequences in the *var* gene database using Megablast. Megablast hits are filtered keeping E-value <1e-2 and capped at maximum 200 hits. The DBLα domain subtype most prevalent among the top 50 hits is determined, and all blast hits are filtered keeping only hits with >95% identity across minimum 200 nucleotides to the DBLα-tag. The name of each filtered hit gene is used to retrieve the encoded domain composition of the hit gene from the domain annotation database ("vardb_GEM_domains.txt").

Predicting the domain composition of the gene from which the DBlα-tag was derived
For each hit gene, the domain type found at each domain position 1-10 (D1-D10) (the DBLα domain is at domain position 2) is logged and the summarized counts for all hit genes are written to a fasta file-specific excel sheet named with the input fasta file name. The counts of different domain types found at same domain position is then processed in an interpretation step, in which the main domain type at each position is called if a type accounts for more than 66% of the total domain count at the position. If no consensus is found, no domain annotation is made. If a consensus is found, the program checks if there is consensus on the domains subtype classification by checking if a domain subtype within the main domain type dominates with more than 66% of the counts. If no domain subtype consensus is reached, only the main domain type is given. For example, if 7 of 10 hit genes encode a DBLβ domain at position D4, and five of these are of the DBLβ12 subtype, the domain is called DBLβ12. If 7 of 10 hit genes encode a DBLβ domain at position D4, and four of these are of the DBLβ12 type, the domain is called DBLβ. All *var* genes (except *var3* not amplified by commonly used DBLα tag primers [3]) contain domains at position D1-D5, but only some have domains at position D6-D10. We included the criteria that annotation of domains at D6-D10 was only called if based on >40% the hit genes, as we found that over-prediction of non-existing domains could be reduced to occur in <10% (data not shown).

Excel result sheet output
Each run of Varia_GEM will result in one excel file containing one sheet per input fasta file as well as one sheet termed "All_samples_summary" containing a summary of all of fata file analyses.
In the fasta-file-specific sheets, the counts of database hits and their domain types at each domain position, is listed in database format. Below this, the predicted consensus domain compositions are written as binary counts in an interpretation section of the sheet both as domain positional and non-positional data along with a calculation of the proportion of genes or transcripts within the input fasta file that encode a given domain type. The consensus domain composition is also written in the fasta file-specific excel sheet as a domain string for each tag (shown in Figure 5 in main manuscript text).

The sheet also contains the *Sample_ID*, *Cluster_size*, the cluster-representative sequence, the most frequent DBLα type among top fifty blast database hits and the *#hits in varDB*.
The "All_samples summary" sheet contains the summarized relative expression level for each main domain type found in position D1-D10 and all main domain types and subtypes for all fasta files analysed. The data is given in a table format allowing subsequent merging with phenotypic or clinical data pertaining to each fasta input file (i.e. patient sample).

An example of the output of both modules can be found in the example directory on the GitHub repository.

Application example

Table S1 shows VARIA_VIP reconstruction of *var* genes from DBLα tag PCR fragments previously generated in [4], supplemental Table S1. The full length sequences of these genes were experimentally defined in the paper and Varia correctly predicts the sequence and the annotation of the genes. Thus, Varia allows the user to design PCR primers to confirm the full-length sequence of exon1, Figure S4. Differences in the annotation is due to how the domains were manually defined in the original publication compared to the pipeline in [5]. Varia was run with default parameters.

**Isolate**	**#hits**	**Sequence Match**	**predicted domains (cluster1)**	**paper domains**
BC12a	2	Yes*	DBLa0.6 CIDRa3.1 DBLb5 DBLd1 CIDRb1	DBLa0.6 CIDRa3.1 DBLb5 DBLg5 DBLd1 CIDRb1
J1a	123	Yes	DBLa0.4 CIDRa6 DBLb5 DBLg10 DBLd6 CIDRb2	DBLa0.4/12 CIDRa6 DBLb5
J1b	4	Yes	DBLa0.6 CIDRa3.2 DBLd1 CIDRb1	DBLa0 CIDRa3.1/2 DBLd1 CIDRb1
J1d	3	Yes	DBLa0.4 CIDRa6 DBLb5 DBLg11 DBLz3 DBLe12	DBLa0.4 CIDRa6 DBLb5 DBLg11/15 DBLz3/6 DBLe12
PCM7a	5	Yes**	DBLa0.5 CIDRa2.9 DBLb5 DBLd1 CIDRb3	DBLa0 CIDRa2.5/9 DBLb5/8 DBLd1 CIDRb1/3
PCM7d	36	Yes	DBLa0.18 CIDRa4 DBLb3 DBLg9	DBLa0.18 CIDRa4 DBLb3/5

Supp. Table 1: Result summary of the Varia prediction versus experimentally validated prediction of var genes from sequence tags. Six sample isolates (from [4]) were run through Varia. For each isolate the table shows: name of isolate; no. of hits found between the tag and database; no. of hits after the identity and length filter; no. of filtered hits reported by the paper; domain structure predicted by Varia; domain structure reported by the paper and whether the predictions match. The graphical output is in Figure S4.
*The annotation of the longest hit had a missing DBLg5 domain. Other files in the clusters annotation had the missing subdomain.
** By default the cluster 1 prediction is used, PCM7a uses cluster 2 here.

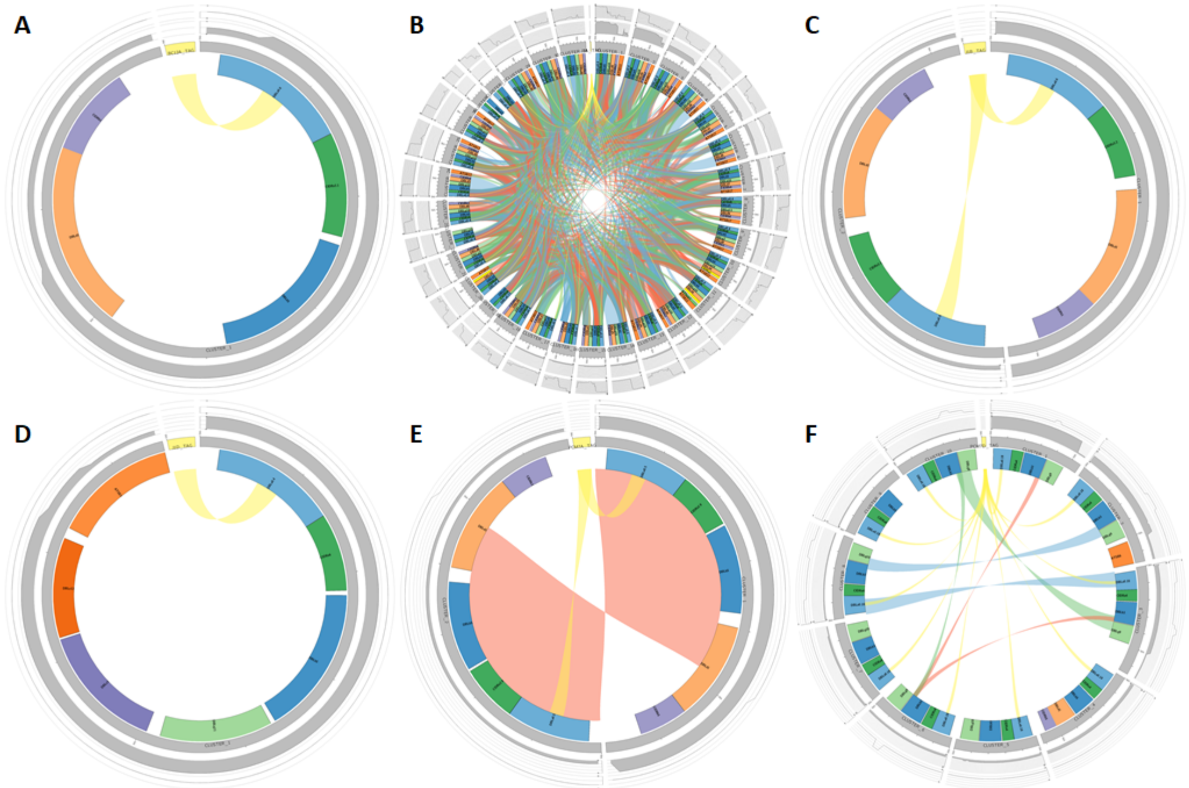

Figure S4. Circos plots from the Varia versus paper prediction comparison: A: BC12a **B:** J1a **C:** J1b **D:** J1d **E:** PCM7a **F:** PCM7d. We can clearly see which of the samples had a higher number of filtered hits than the others in these plots, A and D show only one cluster whilst C and E only have two very similar clusters due to the low number of hits to these isolates.

REFERENCES

1. Morgulis A, Coulouris G, Raytselis Y, Madden TL, Agarwala R, Schaffer AA: **Database indexing for production MegaBLAST searches**. *Bioinformatics* 2008, **24**(16):1757-1764.
2. Rognes T, Flouri T, Nichols B, Quince C, Mahe F: **VSEARCH: a versatile open source tool for metagenomics**. *PeerJ* 2016, **4**:e2584.
3. Lavstsen T, Turner L, Saguti F, Magistrado P, Rask TS, Jespersen JS, Wang CW, Berger SS, Baraka V, Marquard AM *et al*: **Plasmodium falciparum erythrocyte membrane protein 1 domain cassettes 8 and 13 are associated with severe malaria in children**. *Proceedings of the National Academy of Sciences of the United States of America* 2012, **109**(26):E1791-1800.
4. Carrington E, Otto TD, Szestak T, Lennartz F, Higgins MK, Newbold CI, Craig AG: **In silico guided reconstruction and analysis of ICAM-1-binding var genes from Plasmodium falciparum**. *Sci Rep* 2018, **8**(1):3282.
5. Otto TD, Assefa SA, Böhme U, Sanders M, Kwiatkowski D, Pf3k consortium, Berriman M, Newbold C: **Evolutionary analysis of the most polymorphic gene family in falciparum malaria**. *Wellcome Open Res* 2019, **4**(193).
